# miRNAs derived from cobra venom exosomes contribute to the cobra envenomation

**DOI:** 10.1186/s12951-023-02131-7

**Published:** 2023-09-30

**Authors:** Tianci Liao, Mailin Gan, Yanhao Qiu, Yuhang Lei, Qiuyang Chen, Xingyu Wang, Yiting Yang, Lei Chen, Ye Zhao, Lili Niu, Yan Wang, Shunhua Zhang, Li Zhu, Linyuan Shen

**Affiliations:** 1 Farm Animal Genetic Resource Exploration and Innovation Key Laboratory of Sichuan Province, Sichuan Agricultural University, Chengdu, 611130 China; 2 Key Laboratory of Livestock and Poultry Multi-omics, Ministry of Agriculture and Rural Affairs, College of Animal and Technology, Sichuan Agricultural University, Chengdu, 611130 China

**Keywords:** Cobra, Cobra venom, Exosomes, miRNAs, Cobra envenomation

## Abstract

Currently, there is an increasing amount of evidence indicating that exosomes and the miRNAs they contain are crucial players in various biological processes. However, the role of exosomes and miRNAs in snake venom during the envenomation process remains largely unknown. In this study, fresh venom from *Naja atra* of different ages (2-month-old, 1-year-old, and 5-year-old) was collected, and exosomes were isolated through ultracentrifugation. The study found that exosomes with inactivated proteins and enzymes can still cause symptoms similar to cobra envenomation, indicating that substances other than proteins and enzymes in exosomes may also play an essential role in cobra envenomation. Furthermore, the expression profiles of isolated exosome miRNAs were analyzed. The study showed that a large number of miRNAs were co-expressed and abundant in cobra venom exosomes (CV-exosomes) of different ages, including miR-2904, which had high expression abundance and specific sequences. The specific miR-2094 derived from CV-exosomes (CV-exo-miR-2904) was overexpressed both in vitro and in vivo. As a result, CV-exo-miR-2904 induced symptoms similar to cobra envenomation in mice and caused liver damage, demonstrating that it plays a crucial role in cobra envenomation. These results reveal that CV-exosomes and the miRNAs they contain play a significant regulatory role in cobra envenomation. Our findings provide new insights for the treatment of cobra bites and the development of snake venom-based medicines.

**Graphic abstract:**

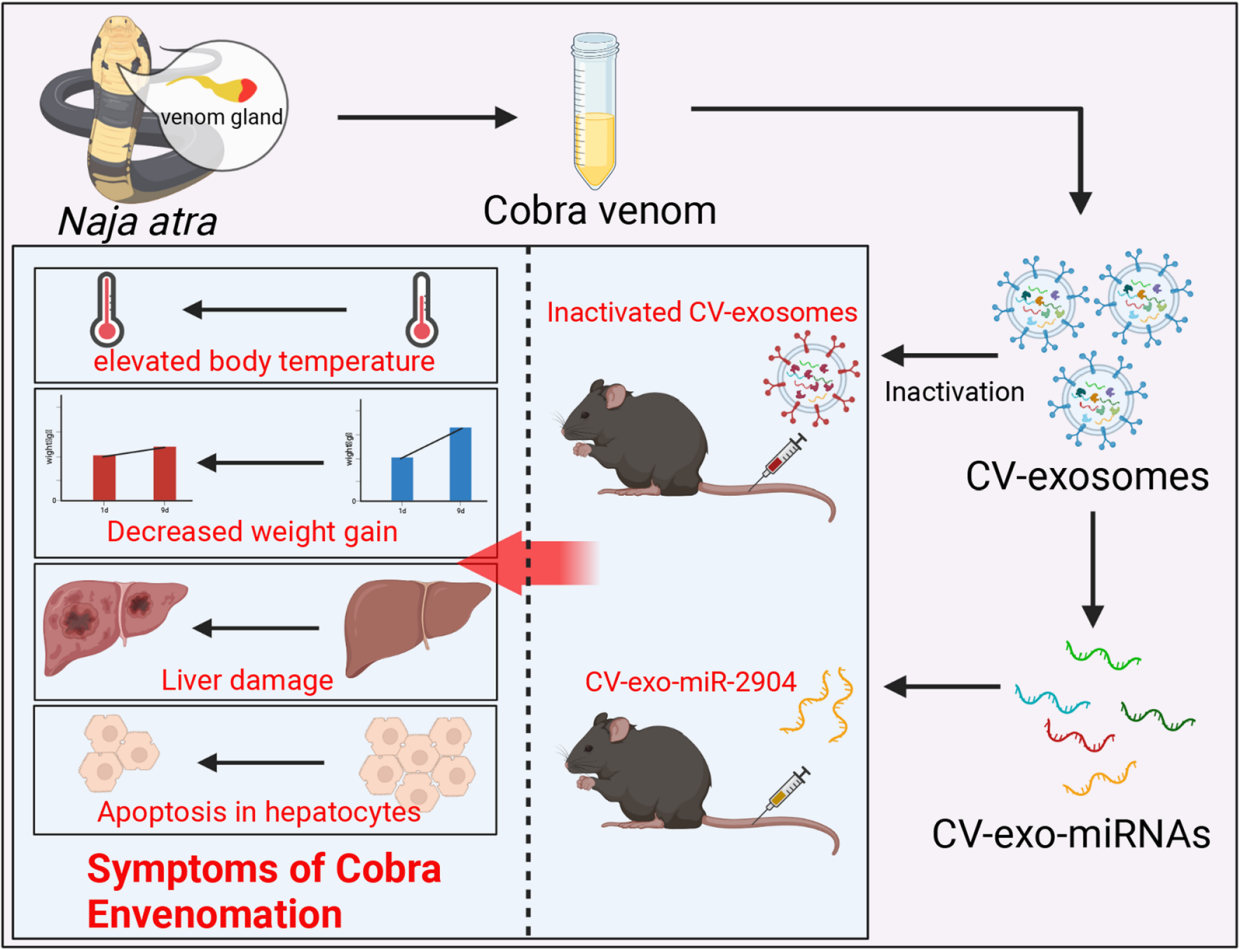

**Supplementary Information:**

The online version contains supplementary material available at 10.1186/s12951-023-02131-7.

## Introduction

Snake envenomation is a common occurrence in tropical regions, with cobra venom being a complex mixture of neurotoxins and blood circulation toxins [1, 2]. The cytotoxin, neurotoxin, phospholipase A2 (PLA2), and other toxic components of cobra venom can lead to a range of clinical symptoms, including inflammation, coagulopathy, neuromuscular dysfunction, and cellular and organ damage [3, 4]. If left untreated, these symptoms can lead to amputation or even death. Exosomes are small vesicles, typically with a diameter of 30–150 nm [5], that release their contents, such as proteins, RNA, and non-coding RNA, to receptor cells or tissues in the extracellular environment, resulting in various biological functions [6, 7]. Yuko et al. were the first to discover exosomes in snake venom and identified a significant number of enzymes and proteins that are related to the biological function of venom, such as PLA2, aminopeptidase A (APA), and dipeptidyl peptidase IV (DPP IV) [8]. This suggests that exosomes are injected into prey along with venom during cobra attacks and may be directly related to the symptoms of snake envenomation. miRNAs have been found to play an essential regulatory role in various biological processes [9, 10], with exosomes carrying a large number of miRNAs that have various regulatory effects on receptor cells and tissues [11, 12]. For instance, studies have demonstrated that miR-31-5p found in milk can greatly enhance endothelial cell function in vitro, stimulate angiogenesis, and promote diabetic wound healing in vivo in mice [13]. Furthermore, miR-423-3p in exosomes can impede the advancement of cervical cancer (CC) cells by hindering macrophage M2 polarization [14]. Currently, most research is concentrated on analyzing the proteins, enzymes, and peptides in snake venom and exosomes, while disregarding the crucial role played by these miRNAs in various biological processes.

Currently, numerous studies indicate that the composition of venom varies among snakes from all taxonomic levels and is influenced by factors such as age, gender, and diet [15, 16]. In this study, we collected venom from three different age groups of male *Naja atra* and successfully isolated exosomes. Surprisingly, we found that even inactivated cobra venom exosomes (CV-exosomes) still caused mice to exhibit signs of cobra envenomation. We then sequenced the miRNAs in these exosomes and found a large number of highly expressed and co-expressed miRNAs in CV-exosomes of different ages. Furthermore, our in vivo and in vitro experiments demonstrated that miRNAs derived from CV-exosomes have similar biological effects to cobra venom. These results provide us with a better understanding of the mechanism of cobra venom and offer new insights for the treatment of snake envenomation and snake venom medicinal research.

## Materials and methods

### Cobra venom taken and exosome extraction and identification

Fresh venom from *Naja atra* was collected from various age groups (2-month-old, 1-year-old, and 5-year-old). The venom was obtained by allowing the snake to bite onto a sterile 50 ml centrifuge tube covered with a latex film, about 20ml venom was extracted from snakes of different ages (at least 20 snakes venoms were extracted in each group). The venom was then mixed with an equal volume of PBS and centrifuged at 2000×g for 10 min at 4℃ using a Fisher Scientific centrifuge. The resulting supernatant was transferred to a new tube and centrifuged at 10,000×g at 4 ℃ for 30 min. The supernatant was then transferred to a new tube and centrifuged at 100,000×g for 70 min at 4 °C. The supernatant was discarded, and the precipitate was re-diluted with PBS, and centrifuged again at 100,000×g at 4℃ for 70 min. Finally, the supernatant was discarded, and the precipitate particles were resuspended in PBS and stored at −80 ℃ for subsequent experiments. The isolated exosomes were identified using atomic force microscopy (AFM). Briefly, the exosome samples are first dispersed in an appropriate buffer and dropped on a liner. Then the sample surface is scanned using a Dimension Icon AFM (Bruker, USA) and the topology and properties of the sample are measured by the interaction forces between the probe and the sample surface. Finally, by analyzing the images obtained by AFM, parameters such as particle size, height, and shape of the exosomes, as well as surface characteristics can be calculated to identify the exosomes.

### Negative staining electron microscopy

The extracted exosomes were placed on a glow-discharged carbon-coated copper grid (Nisshin EM, Japan), and the samples were placed in contact with the grid. The samples were then stained with 2% uranyl acetate at room temperature for 2 min. Excess negative stain was gently blotted away using filter paper, but avoiding excessive blotting to keep the sample on the grid. the grid was examined with an electron microscope (JEM-2100, JEOL).

### miRNAs sequencing

Small RNA sequencing libraries were prepared using the TruSeq small RNA Sample Prep Kits (Ilumina, San Diego, USA). Total RNA was extracted from the samples and the miRNA properties were used, with the 5′ end having a phosphate group and the 3′ end having a hydroxyl group. T4 RNA ligase 2 was used to successively link an adenosine single-stranded DNA 3′ linker and 5’ linker to small RNA. The 5′ end adaptor was designed to capture small RNA with a 5′ phosphate group. The small RNA sequence with a 5′ and 3′ linker was reverse transcribed with a 3-terminal complementary RT primer. Finally, the cDNA sequence produced by reverse transcription was subjected to PCR amplification. The PCR products in the length range of 140–160 bp were recovered by 6% polyacrylamide Tris-borate-EDTA gel to complete the preparation of the entire library. The constructed library was sequenced using Illumina HiSeq 2000/2500, and the sequencing read length was 1 × 50 bp.

### miR-2904 derived from CV-exosomes and CV-exosomes was injected in vivo

In vivo experiments were conducted on male C57BL/6J mice (8-weeks-old) obtained from Dossy, China. Based on previous studies, the optimal therapeutic dosage for exosome injection in mice was found to be 10^5^ particles/g [17]. Thus, exosomes were injected into the mice through tail vein injection according to this standard. Correspondingly, PBS was injected as the negative control (NC) group. To inactivate CV-exosomes, autoclaving was performed at 120 KPa, 121 °C for 30 min. Agomir-2904 was used to overexpress the expression of miR-2904 in mice. A total of 40µL of 10 nM agomir-2904 was administered to the mice through tail vein injection, and the control group was injected with the same amount of agomir nc. All treatment and control groups had at least 3 biological replicates. The nucleotide sequence of agomir used in this study is listed in Additional file 1: Table S1. All animal experiments and procedures were approved by the Research and Animal Ethics Committee of Sichuan Agricultural University (Sichuan, China; NO. DKY-B20131403).

### HE staining

Liver tissue was fixed with 4% paraformaldehyde fixative (Servicebio, China) and was then embedded in paraffin wax. Hematoxylin-eosin staining was performed on liver tissue sections using the Gill II kit (Sigma-Aldrich, USA) modified hematoxylin solution. The operation was performed according to the kit instructions. The liver tissue was cut into slices, soaked in distilled water, and stained with hematoxylin aqueous solution for 5–10 min. The slices were then rinsed with tap water and immersed in acidic alcohol for a few seconds. After rinsing again with tap water for 10 min, the slices were soaked in 1% eosin staining solution for 2–5 min, and then dehydrated in 70%, 90%, and 100% ethanol, followed by xylene. Finally, the slices were mounted with neutral resin and examined under a microscope.

### Total RNA extraction and real-time quantitative PCR

Total RNA (hepatic tissue and HepG2 cell samples) was extracted using the RNAiso reagent (Takara, Japan), following the manufacturer’s instructions. For miRNA reverse transcription, the Mir-X^™^ miRNA First-Strand Synthesis kit (Takara, Japan) was used, while the PrimeScript^™^ RT reagent kit (Takara, Japan) was used for mRNA cDNA synthesis. Real-time quantitative PCR (q-PCR) was carried out using TB Green Premix Ex TaqTM II (Takara, Japan), and gene expression was calculated using the 2^−ΔΔCT^ method. The q-PCR primers used are listed in Additional file 1: Table S2.

### mRNA transcriptome sequencing

Total RNA was extracted from the sample and DNA was removed by DNase treatment. To enrich eukaryotic mRNA, magnetic beads with Oligo(dT) were used, while in the case of prokaryotes, rRNA was removed by a kit to enrich mRNA. The mRNA was fragmented and used as a template to synthesize cDNA using random primers. A double-stranded cDNA synthesis reaction was then performed, where dTTP was replaced by dUTP. Different adaptors were ligated to the cDNA, and a chain containing dUTP was digested by the UNG enzyme method, resulting in the retention of only one strand of cDNA with different adaptors. The purified cDNA strand was repaired at the end, A-tailed, and ligated with sequencing adaptors. The fragment size was selected, and finally, PCR amplification was performed. The constructed library was qualified using an Agilent 2100 Bioanalyzer and sequenced using an Illumina HiSeqTM 2500 or other sequencers.

### Immunofluorescence

Cells were cultured in 12-well plates and fixed with 4% paraformaldehyde (Servicebio, China) for 20 min. Next, they were permeabilized using 0.5% Triton X-100 for 20 min and then blocked with goat serum for 30 min. Primary antibodies against Casepase3 and P53 (ABclone, China) were added at a dilution of 1:100 and incubated overnight at 4 °C. The cells were then incubated with a fluorescent secondary antibody (ABclone, China) at a dilution of 1:100 at room temperature for 1 h. DAPI (ABclone, China) was added in the dark at room temperature for 15 min. Finally, cells were imaged using a fluorescence microscope (Olympus, Japan) and the images were analyzed using Image J software.

### Cell culture and transfection

The hepatocyte line HepG2 was cultured in GM supplemented with DMEM medium (Hyclone), 10% fetal bovine serum, and 1% penicillin-streptomycin (Gibco). RNA oligonucleotides purchased from Yeda (Chengdu, China) were used in this study. RNA oligonucleotides were transfected into HepG2 cells at a final concentration at 20 μm using Lipofectamine 3000 (Invitrogen, USA) according to the manufacturer’s instructions. The cell samples for q-PCR were collected 24 h after transfection. The oligonucleotide sequences used in this study are listed in Additional file 1: Table S1.

### CCK-8 and LDH-cytotoxicity assay

HepG2 cells were cultured in GM and seeded in a 96-well plate at a 30% confluence. The CCK-8 assay kit and LDH-Cytotoxicity Assay Kit, both purchased from Beyotime (Shanghai, China), were used to measure cell viability and cytotoxicity, respectively. The CCK-8 assay was performed at 0, 24, 48, 72, and 96 h after transfection according to the manufacturer’s instructions. The LDH-Cytotoxicity Assay was performed 24 h after transfection following the manufacturer’s instructions. There were at least 3 biological replicates at each time point.

### Biochemical analysis

After the mice were sacrificed, the blood was collected, and then centrifuged at room temperature to separate the serum. Alanine aminotransferase (ALT), aspartate aminotransferase (AST), creatine kinase (CK), cholinesterase (CHe), total bilirubin (TB) and direct bilirubin (DB), and decreased levels of creatinine (CRE) in serum were determined by BS-2000M automatic biochemical analyzer (Mindray, China) according to manufacturer ‘s instructions.

### Flow cytometry

Apoptosis was assessed using flow cytometry on a FACS Canto II flow cytometer (BD Biosciences). The Annexin V-FITC Apoptosis Detection Kit I (BD Pharmingen, USA) was utilized following the manufacturer’s instructions. Briefly, cells were resuspended in 1×  Binding Buffer at a concentration of 1 × 10^6^ cells/ml and added to a tube containing Annexin V-FITC reagent (5 µl) and PI reagent (5 µl). The samples were then incubated for 20 min at room temperature in the dark. Afterward, the samples were analyzed by flow cytometry within 1 h. Compensation and quadrants were set up using the following controls: (1) Unstained cells; (2) Cells stained with FITC Annexin V (no PI); (3) Cells stained with PI (no FITC Annexin V).

### Bioinformatics analysis

Gene Ontology (GO) enrichment analysis and Kyoto encyclopedia of genes and genomes (KEGG) pathway analysis of dysregulated mRNAs and potential target genes of the top 10 significantly different miRNAs between NC and CV-exosome treatment groups and dysregulated mRNAs between agomir nc and CV-exo-miR-2904 agomir treatment groups were performed on the DAVID (https://david.ncifcrf.gov/). Gene set enrichment analysis (GSEA) of dysregulated mRNAs between NC and CV-exosome treatment groups and dysregulated mRNAs between agomir nc and CV-exo-miR-2904 agomir treatment groups were performed on the OECloud tools (https://cloud.oebiotech.cn). We predicted the targeted mRNAs corresponding top-10 miRNAs to use TargetScan (https://www.targetscan.org/) and miRDB (https://www.mirdb.org/). The mRNA interaction network diagram using cytoscape 3.8. If not specified, all steps are performed under the default parameters according to the instructions of the online tool.

### Statistical analysis

The data of continuous normal distribution are expressed as mean ± standard deviation. Analyze data using GraphPad Prism software V.8.0 (GraphPad Software, USA). Two-tailed student t test was used to analyze the differences between groups. *P* < 0.05 was considered statistically significant.

## Results

### Identification of CV-exosomes and injection of inactivated CV-exosomes caused symptoms of cobra envenomation in mice

**Fig. 1:**
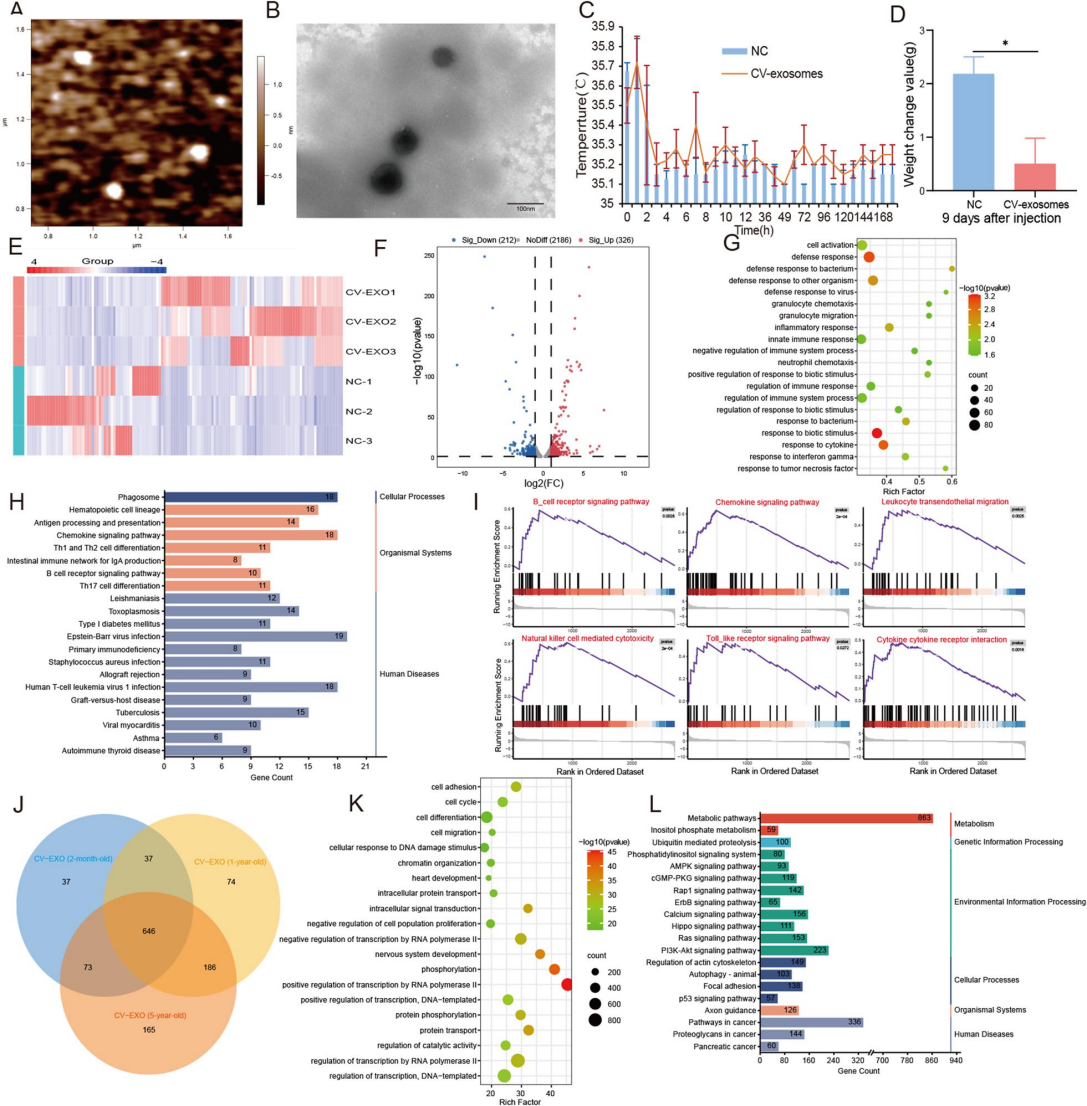
Injection of inactivated CV-exosomes caused symptoms of cobra envenomation in mice and expression of miRNAs in CV-exosomes. **A** The results of CV-exosomes identification by atomic force microscopy. **B** The results of CV-exosomes identification by negative staining electron microscopy. **C** Changes in mice body temperature after injection with CV-exosomes. **D** Changes in mice body weight gain 9 days after CV-exosome injection. **E** Heatmap representation of dysregulated mRNAs in the liver 9 days after CV-exosome injection. **F** Volcano plot showing significantly differentially expressed mRNAs between the NC and CV-exosome treatment groups. **G** GO enrichment analysis of dysregulated mRNAs between NC and CV-exosome treatment groups. **H** KEGG pathway analysis of dysregulated mRNAs between NC and CV-exosome treatment groups. **I** GSEA of dysregulated mRNAs between NC and CV-exosome treatment groups. **J** miRNA content of CV-exosomes from different ages. **K** GO enrichment analysis of the top 10 significantly different miRNAs after CV-exosome treatment. **L** KEGG pathway analysis of the top 10 significantly different miRNAs after CV-exosome treatment. Data are presented as mean ± SD. * represents *P* < 0.05. *NC* negative control

Initially, ultracentrifugation was used to isolate exosomes from *Naja atra* venom, and they were observed and identified using the atomic force microscope and negative staining electron microscopy, demonstrating a successful isolation (Fig. 1A, B). Cobra venom primarily contains proteins and enzymes [18], which are also the main substances in snake venom exosomes [8, 19]. The exosomes were then treated to inactivate all proteins and enzymes before being injected into 8-week-old mice through tail vein injection, with PBS being used as a control. The condition of the mice was observed, with the temperature being measured using an infrared thermometer to prevent stress from affecting the experiment. The results indicated a significant increase in the temperature of mice after injection with CV-exosomes (Fig. 1C). Moreover, the weight gain of mice in the CV-exosome treatment group was found to be significantly lower than that of the control group (Fig. 1D), indicating a significant effect of inactivated CV-exosomes on mice. In addition, serum biochemical indicators revealed increased levels of ALT, AST, CK, LDH, CHe, TB and DB, and decreased levels of CRE in the exosome-treated group (Additional file 1: Table S3), suggesting damage to the muscles, liver, kidney, and bile organs of the mice in the exosome-treated group [20]. After analyzing the changes in these indicators, we observed that they closely resemble the clinical symptoms of cobra envenomation, as reported in previous studies [20]. As the liver plays a crucial role in metabolism, immune function, and detoxification in the human body, previous studies have established its close association with the process of snake venom envenoming [21]. Therefore, we conducted transcriptome sequencing of the liver treated with exosomes and observed a significant change in the transcriptome level. A total of 13,025 genes were identified, out of which 326 genes were significantly up-regulated and 212 genes were significantly down-regulated compared to the control group (|LogFC| > 1, P < 0.05) (Fig. 1E, F). Further GO enrichment analysis showed that the differentially expressed genes were mainly enriched in biological processes such as inflammatory response, immune response, and defense response (Fig. 1G). KEGG pathway analysis revealed that the significantly differentially expressed genes were mainly enriched in immune system functions, including immune cell development and differentiation, antigen processing and presentation, immune system-related diseases and infections, immune cell function and signaling pathways and transplantation and immune responses (Fig. 1H). GSEA analysis indicated that these significantly different genes were mainly enriched in inflammatory pathways, immune pathways, and cytotoxicity-induced natural cell death pathways (Fig. 1I). Overall, these findings suggest that the inactivated CV-exosomes have successfully induced symptoms similar to cobra envenomation in mice. Additionally, our results support the hypothesis that apart from proteins, enzymes, and peptides, CV-exosomes may also contain other non-protein substances, such as miRNAs, that could play a vital role in the process of cobra envenomation.

### Expression of miRNAs in CV-exosomes

In previous reports, it has been shown that in addition to proteins, enzymes, and peptides, exosomes also transport a large number of RNA, such as miRNAs, which play a regulatory role in recipient cells [22, 23]. Therefore, we investigated whether miRNAs present in CV-exosomes have a unique regulatory role in cobra envenomation. We sequenced and analyzed miRNAs in CV-exosomes of three different ages. 793 miRNAs were successfully identified in 2-month-old CV-exosomes, 943 miRNAs were identified in 1-year-old CV-exosomes, and 1070 miRNAs were identified in 5-year-old CV-exosomes. Further analysis showed that 646 miRNAs were co-expressed in the three age groups, accounting for 53.04% of the total identified miRNAs. These co-expressed miRNAs may play an important role in cobra envenomation (Fig. 1J). We further analyzed the 646 miRNAs and identified the top 10 miRNAs with the highest expression abundance (Additional file 1: Table S4). GO enrichment analysis of potential target genes of these top 10 miRNAs revealed that they were mainly involved in biological processes such as cell survival and proliferation, molecular transport and cell signaling, and transcriptional regulation and epigenetics (Fig. 1K). KEGG pathway analysis showed that the potential target genes were mainly enriched in pathways related to cell metabolism, proliferation, and apoptosis, such as Metabolic pathways, p53 signaling pathways, PI3K-Akt signaling pathway, and Hippo signaling pathways (Fig. 1L), which are consistent with the symptoms of cobra envenomation, such as causing tissue necrosis, promoting apoptosis, etc [24, 25]. Further, we compared the sequence of the top 10 miRNAs on NCBI and miRbase (https://www.mirbase.org/) and found that miR-2094 derived from CV-exosomes (CV-exo-miR-2904) is derived from the genome of snake species (Additional file 1: Fig. S1A) and has a specific sequence (Additional file 1: Fig. S1B), which makes it an interesting candidate for further research.

**Fig. 2:**
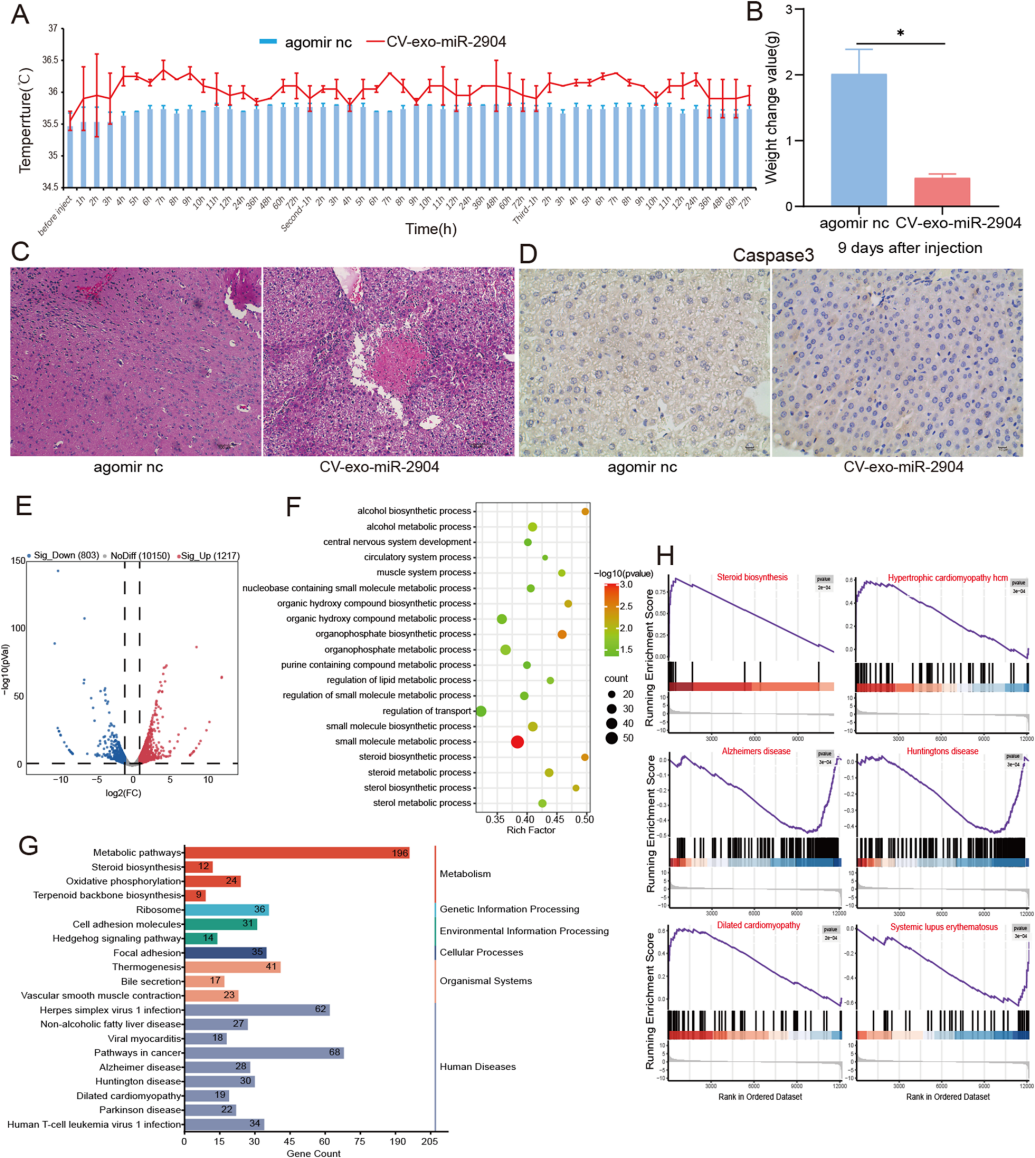
Injection of CV-exo-miR-2904 caused symptoms of cobra envenomation in mice. **A** Changes in mouse body temperature after injection with CV-exo-miR-2904 agomir. **B** Changes in mouse body weight 9 days after injection with CV-exo-miR-2904 agomir. **C** Liver tissue sections stained with hematoxylin and eosin after treatment with CV-exo-miR-2904 agomir. The scale bar represents 100 μm. **D** Immunohistochemical staining for Caspase 3 in liver tissue after injection with CV-exo-miR-2904 agomir. The scale bar represents 10 μm. **E** Volcano plot of significantly differentially expressed mRNAs between the agomir nc and CV-exo-miR-2904 agomir groups. **F** GO enrichment analysis of significantly differentially expressed mRNAs between the agomir nc and CV-exo-miR-2904 agomir groups. **G** KEGG pathway analysis of significantly differentially expressed mRNAs between the agomir nc and CV-exo-miR-2904 agomir groups. **H** GSEA of differentially expressed mRNAs between the agomir nc and CV-exo-miR-2904 agomir groups. Data are presented as mean ± SD. * represents P < 0.05. *agomir nc* agomir negative control

### Injection of CV-exo-miR-2904 caused symptoms of cobra envenomation in mice

**Fig. 3:**
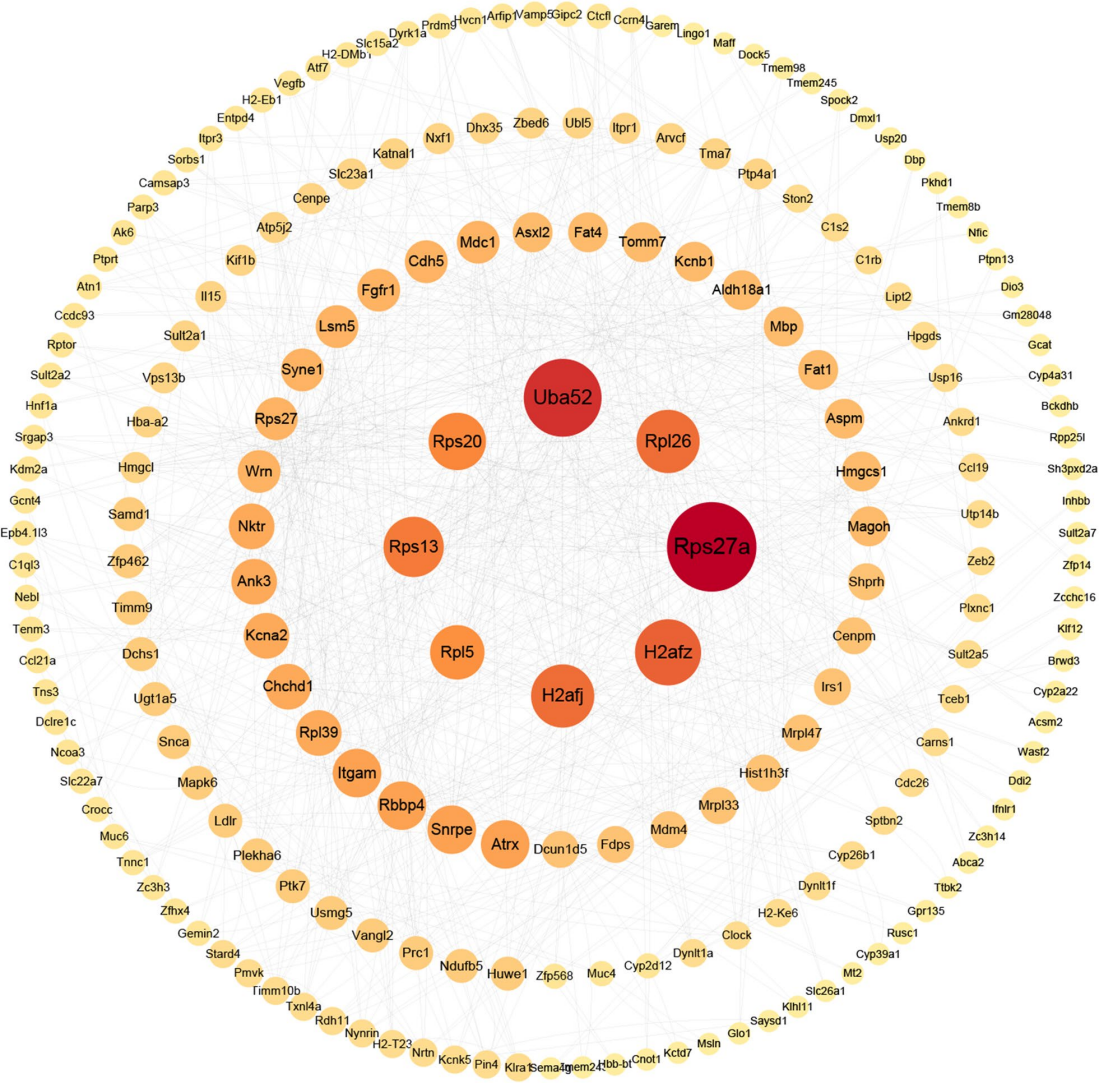
PPI networks reveal significantly different genes after CV-exo-miR-2904 agomir injection and their involved biological processes. The PPI network diagram was created using Cytoscape v3.8.0 software

In the above research, it was demonstrated that even inactivated CV-exosomes can still cause symptoms similar to cobra envenomation in mice. To investigate the role of miRNAs in cobra envenomation, we synthesized the CV-exo-miR-2904 agomir and injected it into mice via tail vein injection, and the control group was injected with agomir nc. The temperature and weight of the mice were monitored after injection, and the results showed a significant increase in temperature (Fig. 2A) and a significant decrease in weight gain after 9 days (Fig. 2B). The serum biochemical indicators showed an increase in ALT, AST, CK, LDH, CHe, TB and DB and a decrease in CRE in the CV-exo-miR-2904-treated mice, indicating organ damage to the liver, kidney, muscle, and bile (Additional file 1: Table S5). HE staining of the liver of mice treated with CV-exo-miR-2904 showed significant edema in the liver tissue (Fig. 2C). Additionally, the results of immunohistochemistry showed a significant increase in the expression of Caspase3 in the liver tissue, suggesting an aggravation of apoptosis (Fig. 2D). Furthermore, transcriptome sequencing of the liver of CV-exo-miR-2904-treated mice identified 12,170 genes, of which 1217 genes were significantly up-regulated and 803 genes were significantly down-regulated (|LogFC| > 1, P < 0.05) (Fig. 2E). Interestingly, GO enrichment analysis of the significantly affected genes were found to be mainly involved in metabolic biological processes occurring in the liver, such as alcohol, steroids, sterols, nucleobase-containing small molecules, purine compounds, and cholesterol (Fig. 2F). KEGG pathway analysis revealed that the significantly different genes are mainly enriched in pathways related to metabolism, cellular structure and function, as well as disease and neural pathways (Fig. 2G). GSEA analysis of these gene sets revealed that the differential genes were mainly enriched in pathways related to neurological diseases, immune diseases, heart diseases, and steroid biosynthesis (Fig. 2H). Subsequently, based on the significantly differentially expressed genes, we constructed a protein-protein interaction (PPI) network map. Through the analysis of network topology and node properties, we found that there were highly centralized central nodes in the network, such as Uba52, Rps27a, Rpl26, Rps20, Rps13, Rpl5, H2afj, and H2afz. These node proteins play a crucial role in protein synthesis, regulating the stability, function, and localization of proteins, as well as regulating DNA modification and mRNAs transcription. In addition, these proteins are also closely related to the biogenesis of miRNAs, and the proliferation, differentiation, and apoptosis of cells (Fig. 3). These findings indicate that direct injection of CV-exo-miR-2904 leads to organ damage in mice, particularly in the liver tissue. Notably, these symptoms are highly consistent with the clinical symptoms observed in cases of cobra envenomation [20, 24], suggesting that CV-exo-miR-2904 induces cobra envenomation-like symptoms in mice.

### CV-exo-miR-2904 inhibits hepatocytes proliferation and promotes apoptosis in vitro

**Fig. 4:**
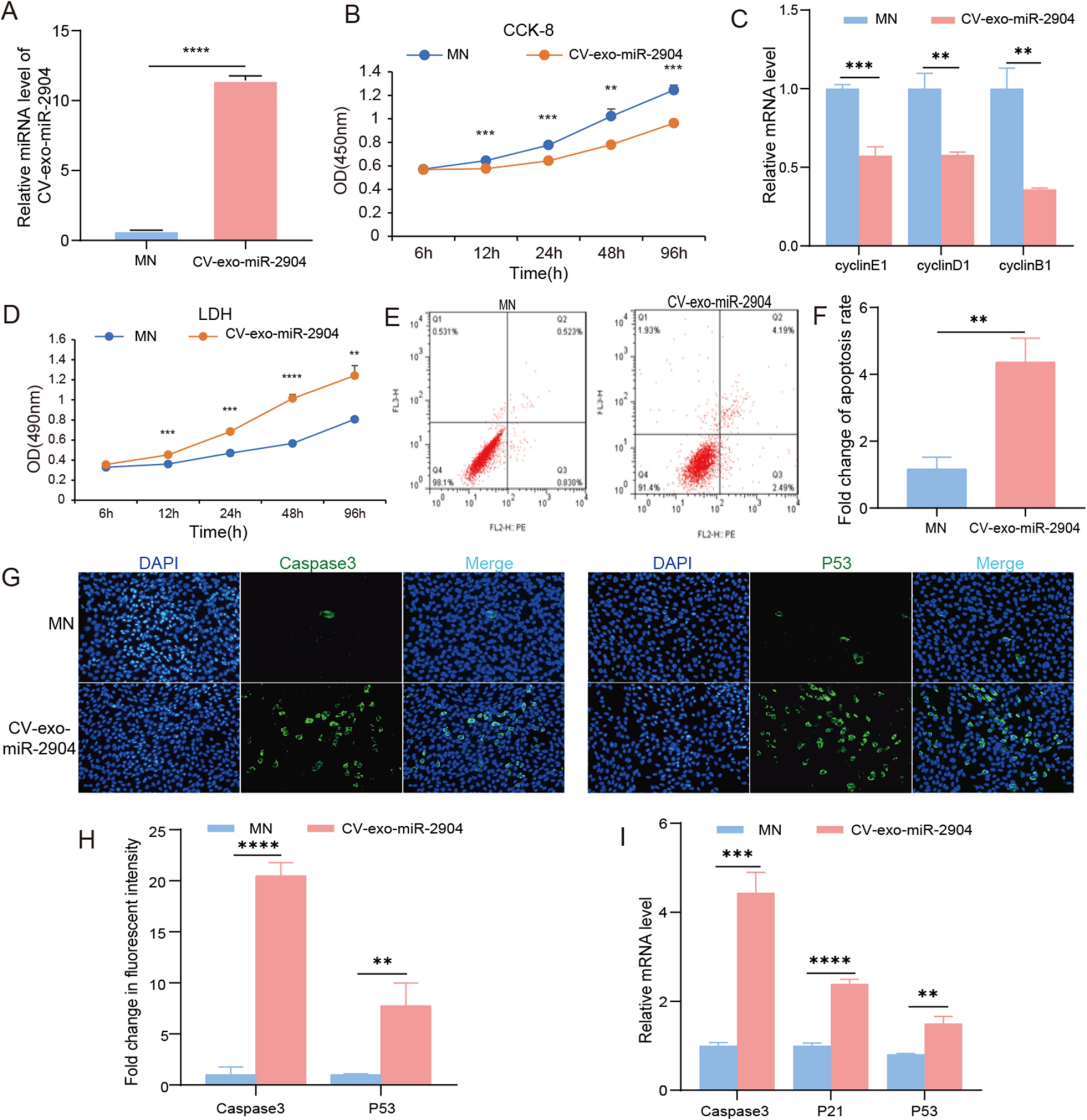
CV-exo-miR-2904 inhibits hepatocytes proliferation and promotes apoptosis in vitro. **A** Expression of CV-exo-miR-2904 in HepG2 cells after overexpression. **B** CCK-8 assay to determine the effect of CV-exo-miR-2904 on the viability of HepG2 cells. **C** The relative mRNA expression of proliferation marker genes, cyclinE1, cyclinD1, and cyclinB1, after CV-exo-miR-2904 overexpression in HepG2 cells. **D** LDH-cytotoxicity assay to detect the effect of CV-exo-miR-2904 overexpression on the cytotoxicity of HepG2 cells. **E** Flow cytometry results to assess the effect of CV-exo-miR-2904 overexpression on apoptosis. **F** Quantification of flow cytometry results: Cell apoptosis rate = early cell apoptosis rate (Q2) + late cell apoptosis rate (Q3). **G** Immunofluorescence staining of apoptosis-related proteins, Caspase3 and P53, in HepG2 cells overexpressing CV-exo-miR-2904. Scale: 1 bar represents 100 μm. **H** The amount of Caspase3 and P53 was quantified using the ImageJ software. **I** The relative mRNA expression of apoptosis markers, Caspase3, P21, and P53, after CV-exo-miR-2904 overexpression in HepG2 cells. Data are presented as mean ± SD. ** represents *P* < 0.05, *** represents *P* < 0.001, and **** represents *P* < 0.0001. *MN* mimic negative control

In this study, the effect of CV-exo-miR-2904 on liver organs in mice was found to be particularly significant. To further verify its effect, the expression level of CV-exo-miR-2904 was overexpressed in the HepG2 hepatocyte line in vitro, mimic nc (MN) as a control group (Fig. 4A). The CCK-8 cell viability assay kit was used to detect the cell viability of HepG2 cells after overexpression of CV-exo-miR-2904, and the results showed that CV-exo-miR-2904 significantly inhibited the viability of HepG2 cells, indicating that it can inhibit the proliferation of HepG2 cells (Fig. 4B). Additionally, CV-exo-miR-2904 also inhibited the mRNA expression levels of proliferation marker genes, such as cyclinE1, cyclinD1, and cyclinB1 (Fig. 4C). LDH-cytotoxicity analysis kit was used to detect the effect of CV-exo-miR-2904 overexpression on the cytotoxicity of HepG2 cells, and it was found that CV-exo-miR-2904 promoted the release of cytotoxicity, indicating that overexpression of CV-exo-miR-2904 promoted apoptosis (Fig. 4D). The subsequent flow cytometry results also supported this observation (Fig. 4E, F). Immunofluorescence staining of apoptosis-related proteins Casepas3 and P53 in cells overexpressing CV-exo-miR-2904 was performed, and the results directly proved that CV-exo-miR-2904 could promote the expression of apoptotic proteins (Fig. 4G, H). Finally, q-PCR results also confirmed that CV-exo-miR-2904 can promote the mRNA level of apoptotic markers, such as caspase3, p21, and p53 (Fig. 4I). Overall, in vitro overexpression of CV-exo-miR-2904 inhibited hepatocyte proliferation and promoted apoptosis.

## Discussion

Exosomes are known to contain a diverse range of bioactive substances that are involved in various biological processes, such as intercellular material and information transmission, immune response, antigen presentation, as well as protein and RNA transport [26, 27]. Among these substances, exosomes are known to carry a large quantity of specific RNA, with mature miRNA being the most highly expressed, accounting for approximately 41.72% of the total RNA [28]. In this study, we isolated exosomes from cobra venoms of varying ages and analyzed the miRNA expression profiles. Our findings revealed a substantial number of co-expressed and highly abundant miRNAs in CV-exosomes of different ages. The top 10 miRNAs with the highest expression levels were annotated and enriched in pathways associated with metabolism, cell proliferation, and apoptosis. Previous studies have reported that clinical symptoms following cobra envenomation include metabolic damage and apoptosis of tissues and organs [29–31]. These findings suggest that miRNAs present in CV-exosomes may play a role in the pathophysiology of cobra envenomation. Further analysis revealed that CV-exo-miR-2904 is a unique miRNA of cobra with a specific sequence. Thus, the researchers conducted in vivo and in vitro experiments to verify the biological function of CV-exo-miR-2904. Before this, inactivated CV-exosomes were injected into mice via tail vein injection. Cobra venom contains numerous proteins and enzymes, such as PLA2, APA, and DPP IV, which are crucial in the biological function of cobra envenomation [32, 33]. However, even the inactivated CV-exosomes still caused symptoms of cobra envenomation in mice, such as elevated body temperature, slow weight gain, and abnormal biochemical indicators [21]. Furthermore, transcriptome analysis of the liver revealed a large number of differentially expressed genes, primarily related to cellular immunity, inflammation, and cellular defense response. The liver serves as the primary metabolic, detoxification, and immune organ in animals [34, 35]. These results suggest that inactivated CV-exosomes cause liver damage in mice and produce symptoms similar to those of cobra envenomation. In addition, studies have shown that substances other than snake venom-related proteins and enzymes present in the exosomes of cobra venom may also cause symptoms of cobra envenomation in mice.

Based on the above research, we conducted further experiments to verify whether miRNAs in exosomes play a role similar to snake venom, in addition to proteins and enzymes. Therefore, we synthesized CV-exo-miR-2904 and injected it into mice via tail vein injection. As expected, overexpression of CV-exo-miR-2904 also increased body temperature, decreased weight gain, and resulted in abnormal biochemical indicators in mice, which was consistent with the symptoms after cobra envenomation. Furthermore, liver sections and immunohistochemical results of CV-exo-miR-2904 treated mice more directly proved that overexpression of CV-exo-miR-2904 could lead to edema and apoptosis in liver tissues of mice, which also was consistent with the clinical symptoms after cobra envenomation. Transcriptome sequencing of liver tissue also found a large number of significantly different genes after CV-exo-miR-2904 treatment, and these genes were mainly enriched in nerve, heart, and metabolic functions, especially the biological metabolic function of liver tissue. In order to further study the effect of CV-exo-miR-2904, we overexpressed it in hepatocytes in vitro. The results showed that CV-exo-miR-2904 could inhibit the proliferation of hepatocytes and promote apoptosis. Our study demonstrated that CV-exo-miR-2904 can indeed cause symptoms similar to those after cobra envenomation in mice, especially liver tissue damage.

## Conclusion

Overall, our study has demonstrated that, in addition to proteins, enzymes, and peptides, miRNAs in CV-exosomes also play a critical regulatory role in the process of cobra envenomation. These findings provide a novel perspective for the treatment of cobra envenomation and the development of cobra venom medicine.

### Supplementary Information

**Additional file1 of miRNAs derived from cobra venom exosomes contribute to the cobra envenomation: Figure S1. A** The Nucleotide Blast results of CV-exo-miR-2904 sequence (GCCTCGGTGGGCCTCGGATAGCCG) on NCBI. **B** Results of sequence alignment of CV-exo-miR-2904 on miRbase. **Table S1.** The synthesized RNA oligonucleotides. **Table S2.** The primers used for qRT-PCR. **Table S3.** Biochemical indicators after CV-exosomes treatment.**Table S4.** Top10-miRNAs with the highest expression in exosomes. **Table S5.** Biochemical indicators after CV-exo-miR-2904 treatment.

## Data Availability

The data in this work are available in the manuscript or available from the corresponding author upon reasonable request.

## Abbreviations

CV-exosomes: Cobra venom exosomes
CV-exo-miR-2904: miR-2094 derived from CV-exosomes
PLA2: Phospholipase A2
APA: Aminopeptidase A
DPP IV: Dipeptidyl peptidase IV
AFM: Atomic force microscopy
NC: Negative control
CC: Cervical cancer
GO: Gene Ontology
KEGG: Kyoto encyclopedia of genes and genomes
GSEA: Gene set enrichment analysis
ALT: Alanine aminotransferase
AST: Aspartate aminotransferase
CK: Creatine kinase
Che: Cholinesterase
TB: Total bilirubin
DB: Direct bilirubin
CRE: Creatinine
PPI: Protein–protein interaction
