# Supplementary material for: miRNAs derived from cobra venom exosomes contribute to the cobra envenomation
Source: J Nanobiotechnology. 2023 Sep 30;21:356. doi: 10.1186/s12951-023-02131-7 (PMC10544165; doi:10.1186/s12951-023-02131-7)
Supplement: Supplementary file 1 — Additional file1 of miRNAs derived from cobra venom exosomes contribute to the cobra envenomation: Figure S1. A The Nucleotide Blast results of CV-exo-miR-2904 sequence (GCCTCGGTGGGCCTCGGATAGCCG) on NCBI. B Results of sequence alignment of CV-exo-miR-2904 on miRbase. Table S1. The synthesized RNA oligonucleotides. Table S2. The primers used for qRT-PCR. Table S3. Biochemical indicators after CV-exosomes treatment.Table S4. Top10-miRNAs with the highest expression in exosomes. Table S5. Biochemical indicators after CV-exo-miR-2904 treatment. [file 12951_2023_2131_MOESM1_ESM.pdf]

Additional file:

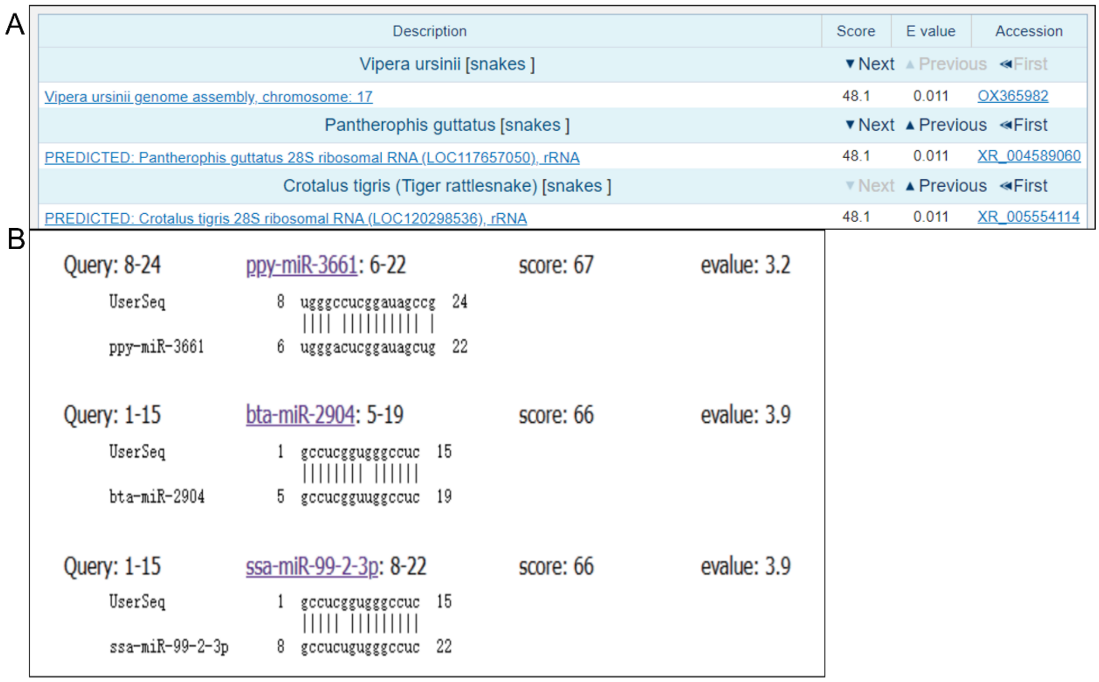

Figure S1. (A) The Nucleotide Blast results of CV-exo-miR-2904 sequence (GCCTCGGTGGGCCTCGGATAGCCG) on NCBI. (B) Results of sequence alignment of CV-exo-miR-2904 on miRbase.

Table S1. the synthesized RNA oligonucleotides.

Targets	Sequence 5'-3'
Mimic nc	UUGUACUACACAAAAGUACUG
miR-2904 mimic	GCCUCGGUGGGCCUCGGAUAGCCG
agomir nc	UUGUACUACACAAAAGUACUG
agomir-2904	GCCUCGGUGGGCCUCGGAUAGCCG

Table S2. The primers used for qRT-PCR.

Targets	primer	Sequence 5'-3'
CV-exo-miR-2904	Forward primer	GCCTCGGTGGGCCTCGGATAGCCG
	Reverse primer	R-Uni-miR qPCR primer, included in kit (miRNA universal downstream primer, TaKaRa)
U6	Forward primer	CTCGCTTCGGCAGCACA
	Reverse primer	AACGCTTCACGAATTTGCGT
β-actin	Forward primer	GGCTGTATTCCCCTCCATCG
	Reverse primer	CCAGTTGGTAACAATGCCATGT
cyclinE1	Forward primer	GTGGCTCCGACCTTTCAGTC
	Reverse primer	CACAGTCTTGTCAATCTTGGCA
cyclinD1	Forward primer	GCGTACCCTGACACCAATCTC

	Reverse primer	CTCCTCTTCGCACTTCTGCTC
cyclinB1	Forward primer	AAGGTGCCTGTGTGTGAACC
	Reverse primer	GTCAGCCCCATCATCTGCG
Caspase3	Forward primer	GAGCTTGGAACGGTACGCTA
	Reverse primer	CCGTACCAGAGCGAGATGAC
P21	Forward primer	CCAAGCCATTCCATAGGCGT
	Reverse primer	AAGGGACATGTGGCTCATCG
P53	Forward primer	CAGTGGGAACCTTCTGGGAC
	Reverse primer	TGAGTGGATCCTGGGGATTGT

Table S3. Biochemical indicators after CV-exosomes treatment.

Item	NC	CV-exosomes	
**ALT**	44	49.5	U/L
**AST**	103.5	122.6	U/L
**BUN**	8.52	7.55	mmol/L
**CRE**	86	33	umol/L
**UA**	247.8	277.2	umol/L
**TB**	0.7	1	umol/L
**DB**	0.5	0.7	umol/L
**TP**	55	59.3	g/L
**ALB**	35.9	36.2	g/L
**GLO**	19.1	23.1	g/L
**A/G**	1.88	1.57	
**CK**	367.5	410.1	u/L
**LDH**	328.5	321.9	u/L
**CHe**	449.7	467.4	u/L

Table S4. Top10-miRNAs with the highest expression in exosomes.

Top10-miR_name	miR_seq	Length	CV1	CV2	CV3	Expression level
miR-99a-5p	AACCCGTAGATCCGATCTTGT	21	8936.78	2634.42	2742.46	high
miR-125b-5p	TCCCTGAGACCCTAACTTGTGA	22	8812.59	4684.14	3084.86	high
miR-200a	TAACACTGTCTGGTAACGATGTT	23	8270.70	4469.36	4879.34	high
miR-6240	CGGCGGGTGTTGACGCGATG	20	7138.52	12114.08	6822.33	high
miR-125a-5p	TCCCTGAGACCCTTAACCTGT	21	6381.80	2154.47	1089.06	high
miR-375-3p	TTTGTTCGTTCGGCTCGCGTT	21	4917.71	7367.62	7298.38	high
miR-2904	GCCTCGGTGGGCCTCGGATAGCCG	24	4911.42	3425.94	1767.67	high
miR-148a-3p	TCAGTGCACTACAGAACTTTCT	22	4679.66	1884.00	1950.41	high
miR-191-5p	CAACGGAATCCCAAAAGCAGCT	22	4403.88	1601.60	1318.43	high
miR-99b-5p	AACCCGTAGATCCGAACTTGCG	22	4086.80	1195.90	906.73	high

Table S5. Biochemical indicators after CV-exo-miR-2904 treatment.

Item	agomir nc	CV-exo-miR-2904	
**ALT**	44	171	U/L
**AST**	103.5	177	U/L
**BUN**	8.52	16.72	mmol/L
**CRE**	86	44	umol/L
**UA**	247.8	114	umol/L
**TB**	0.7	63.54	umol/L
**DB**	0.5	10.35	umol/L
**TP**	55	60.6	g/L
**ALB**	35.9	26.6	g/L
**GLO**	19.1	34.3	g/L
**A/G**	1.88	0.77	
**CK**	367.5	2511	u/L
**LDH**	328.5	836	u/L
**CHe**	449.7	2751	u/L
